# Acute Kidney Injury Associated with Novel Anticancer Therapies: Immunotherapy

**DOI:** 10.34067/KID.0000000749

**Published:** 2025-02-24

**Authors:** Sabine Karam, Ala Ali, Winston Fung, Prashant Mehta, Sanjeev Nair, Urmila Anandh

**Affiliations:** 1 Division of Nephrology and Hypertension, Department of Medicine, University of Minnesota, Minneapolis, Minneapolis; 2 Division of Nephrology and Hypertension, Department of Internal Medicine, American University of Beirut, Beirut, Lebanon; 3 Nephrology and Renal Transplantation Center, The Medical City, Baghdad, Iraq; 4 Department of Medicine and Therapeutics, Prince of Wales Hospital, The Chinese University of Hong Kong, Hong Kong, China; 5 Department of Hematology, Medical Oncology and Bone Marrow Transplantation, Amrita Institute of Medical Sciences and Research Centre, Faridabad, India; 6 Department of Nephrology, Madras Medical Mission, Chennai, India; 7 Department of Nephrology, Amrita Institute of Medical Sciences and Research Centre, Faridabad, India

**Keywords:** AKI, clinical nephrology, drug nephrotoxicity

## Abstract

The landscape of cancer survival has been positively affected by the introduction and dissemination of immunotherapy with the wide usage of immune checkpoint inhibitors and chimeric antigen receptors cell therapies. The success of these novel therapies can, however, be limited to a certain extent by systemic inflammatory toxicities affecting, directly or indirectly, the kidney. In the case of immune checkpoint inhibitors, severe acute interstitial nephritis is the main adverse event and can lead to permanent discontinuation of the therapy. In turn, chimeric antigen receptor cell therapy can cause cytokine release syndrome and immune effector cell-associated hemophagocytic lympho-histiocytosis, with kidney damage through various mechanisms, and be life threatening. Prompt diagnosis and management of these entities is essential to preserve kidney function and ensure the best possible kidney and overall outcomes to patients with cancer.

## Introduction

The landscape of cancer survival has been revolutionized by the linkage of the immune system to oncology and the introduction of immunotherapy with the wide usage of immune checkpoint inhibitors (ICIs) and chimeric antigen receptors cell therapies (CAR-Ts).^1^ In the case of ICIs, there has been achievement of durable remission in a subset of patients with metastatic disease, whereas CAR T-cell therapies have been astoundingly effective in hematologic malignancies, such as acute B-lymphoblastic leukemia and B-cell lymphoma, and are increasingly being used to treat multiple myeloma while being trialed for solid tumors, such as hepatocellular carcinoma and lung cancer, with variable sucess.^2–6^ Other immune cells, such as natural killer cells and macrophages, have also been engineered for cancer immunotherapy of solid tumors because of their ability to penetrate the tumor microenvironment and are being tested in various clinical trials.^6,7^ The success of these novel therapies can, however, be limited to a certain extent by inflammatory toxicities. In the case of ICIs, consistent with the name, they are referred to as immune-related adverse events and can affect different organs and, notably, the kidney with AKI and electrolyte disturbances. This can lead to treatment delays and discontinuation.^8^ In the case of CAR-Ts, systemic hyperinflammatory disorders can arise, notably cytokine release syndrome (CRS) and immune effector cell-associated hemophagocytic lympho-histiocytosis (IEC-HLH), and cause kidney damage through various mechanisms.^9^ In this article, we aim to detail the mechanism of action of these novel therapies, along with the diagnosis and management of their kidney adverse effects. Traditional immunotherapies, such as IFN-*α* and high-dose IL-2, that have been in use for decades and have well-known adverse kidney effects, but are increasingly less used due to systemic toxicity and limited efficacy, will not be addressed.

## Immune ICIs

### Mechanisms of Action

**Figure 1:**
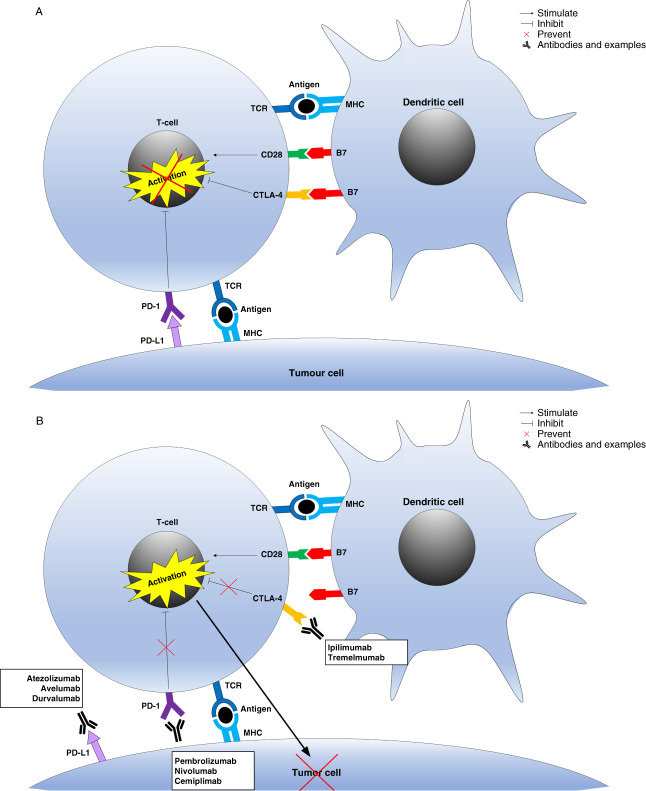
**Immune checkpoint tumor escape mechanisms and ICI mechanism of action.** (A) Immune-mediated tumor escape mechanisms. (B) ICIs’ mechanisms of action. CTLA-4, cytotoxic T-lymphocyte associated protein 4; ICI, immune-checkpoint inhibitors; PD-1, programmed cell death protein 1; PD-L1, programmed death-ligand 1, TCR, T-cell receptor.

Immune checkpoints are a type of costimulatory or coinhibitory molecules expressed on the surface of immune cells, which can regulate effector T-cell responses to modulate the degree of immune activation, a mechanism useful to avoid autoimmunity.^10,11^ Tumor cells usually present antigens that are susceptible to recognition by the immune system, but have developed a repertoire of mechanisms involving these immune checkpoints to evade the anticancer immune response.^12^ ICIs overcome this escape mechanism by blocking the interaction of such immune checkpoints in the tumor cells with some immune cells, preventing activation of the T-cell’s inhibitory signals. This allows the tumor-reactive T-cells to mount an effective anticancer response (Figure 1).^10,11^ There are currently three well-studied immune targets with good clinical data and approved therapeutic agents that are widely used: cytotoxic T-lymphocyte–associated protein 4 (CTLA-4), programmed death-1 protein (PD-1), and programmed death-1 ligand.^10,11^ CTLA-4 is an inhibitory receptor belonging to the CD28 Ig subfamily, which is constitutively expressed on the surface of immunosuppressive T-regulatory cells. In the case of naïve T-cells, CTLA-4 is localized to intracellular vesicles cell and only expressed on the cell surface after their activation.^13^ CTLA-4 induces inhibitory immunomodulation through two mechanisms: extrinsically through competitive binding with the costimulatory molecule CD28 for the B7 ligands, for which CTLA4 has high affinity, and intrinsically by several mechanisms, such as affecting T-cell motility.^14^ Through its competition with CD28, there is CTLA-4–mediated transendocytosis of CD80 and CD86, with subsequently tolerogenic effects on the interacting cell.^15^ mAb targeting CTLA-4, such as ipilimumab and tremelmumab, bind to CTLA-4 in the B7 interaction domain, blocking the binding of CTLA-4 to B7 and preventing T-cell tolerance to the tumor. PD-1 is a coinhibitory receptor, mainly expressed on activated T-cells, B cells, and natural killer cells, whereas PDL-1 is its dominant ligand and is present in multiple cell types, including tumor cells.^16^ The binding of PD-1/programmed death-1 ligand acts primarily to dampen T-cell activation in the periphery and in the inhibition of previously activated T-cells, through the recruitment of Src homology two domain-containing tyrosine phosphatase 2 to suppress T-cell receptor (TCR) signaling transduction.^17^ mAb blocking either PD-1 (pembrolizumab, nivolumab, and cemiplimab) or its ligand PDL-1 (atezolizumab, avelumab, and durvalumab) attenuate these TCR signaling, preventing tumor immune escape through this pathway. The different types of approved ICIs and their current clinical indications are summarized in Supplemental Table 1. Recently, additional immune checkpoint molecular targets have been identified and include T-cell immunoreceptor with Ig and immunoreceptor tyrosine-based inhibitory motif domains, lymphocyte-activation gene-3, T-cell Ig and mucin-domain containing-3 V-domain Ig suppressor of T-cell activation, B-lymphocyte and T-lymphocyte attenuator, and signal regulatory protein *α*.^18^ They have multiple ligands, with complex mechanisms for inducing immunomodulation and interactions between these ligands.^11^ Their therapeutic potential is currently under investigation.

### ICI-AKI: Mechanisms and Types

**Figure 2:**
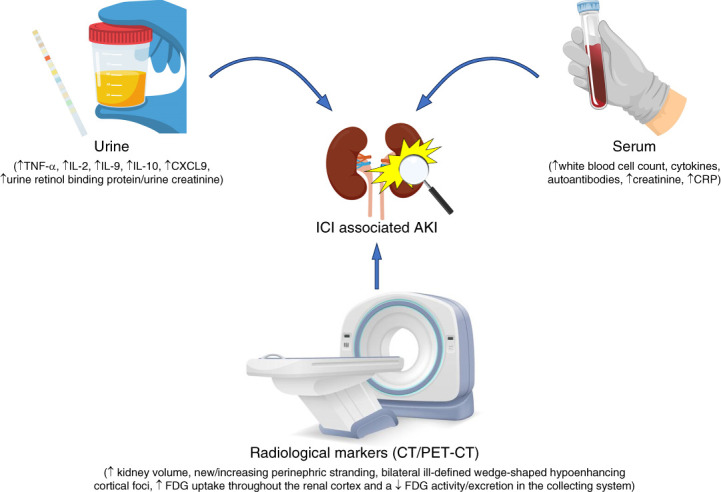
**Noninvasive modalities for the diagnosis of ICI-AKI.** CRP, C-reactive protein; CXC, conserved cysteine motif; CXCL9, CXC motif ligand 9; FDG, fluoro-d-glucose; PET-CT, positron emission tomography–computed tomography; TNF, tumor necrosis factor.

Acute tubulo-interstitial nephritis (ATIN) is the main kidney-related adverse effect of ICI and represents more than 80%–90% of the lesions seen on kidney biopsy in two large multicenter cohorts.^19,20^ Other tubulopathies, manifested by electrolyte abnormalities and renal tubular acidosis, can occur as well.^21,22^ Glomerular lesions have also been described with pauci-immune GN, podocytopathies, and C3 glomerulopathy, the most frequently reported.^23^ The different types of pathologies seen in ICI-AKI are summarized in Figure 2.

The exact mechanism of ATIN is not known. Five proposed mechanisms may interplay:^8^

1. The development and proliferation of autoreactive T-cells and loss of self-tolerance.
2. The development of autoantibodies against antigens that are present on tubular epithelial cells, mesangial cells, and podocytes.
3. Expression of checkpoint receptors on nontumor tissues.
4. ICI-induced reactivation of drug-specific T-cells.
5. Inflammatory renal injury through increased production and elaboration of proinflammatory cytokines.

#### Epidemiology of ICI-AKI

The incidence of ICI-induced AKI varies across the literature. This variation may be related to the study design, region, patient demographics, comorbidities, type of ICI, single versus dual ICI use, AKI definition, and combination with other medications. In addition, the fact that in most instances, causality is inferred rather than biopsy proven makes the estimation more difficult. In a meta-analysis of 48 randomized controlled trials, the pooled estimated incidence rate of AKI during PD-1 inhibitor exposure was 2.2%.^24^ In real life, this risk seems to be slightly higher, as two recent systematic reviews and meta-analyses reported the incidence of ICI-induced AKI to be 3.5% and 5.7%, respectively.^25,26^ Several risk factors for ICI-induced AKI have been described, including preexisting CKD, diabetes, and concomitant extrarenal immune-related adverse events. AKI is also potentially more prevalent in cancer patients receiving ICI therapy if used concomitantly with proton pump inhibitors (PPIs), nonsteroidal anti-inflammatory drugs, renin-angiotensin system inhibitors and blockers, and diuretics.^25–27^ The risk of ICI-induced AKI is higher with anti–CTLA-4 than with anti–PD-1.^28^ The nonspecific action of anti–CTLA-4 in the early stages of T-cell activation is a possible explanation for such a higher incidence.^29^ Consequently, when compared with other ICIs, ipilimumab was reported to be associated with the highest risk of AKI.^21^ Combining anti–CTLA-4 and anti–PD-1 will further the risk in a time and dose-dependent pattern. The higher the target concentration, the more the adverse events.^30^ The median time from ICI initiation to AKI in general is 14–16 weeks, but can range from 6.5 to 21 weeks.^8,31^ Such a late presentation may be related to the long *t*_1/2_ of ICIs, the relatively late recognition of milder forms of AKI, and the insensitivity of creatinine as a marker of AKI.^30,32^ The estimated incidences of ICI-AKI related to the most common classes and the clinical characteristics are presented in Table 1.

**Table 1:** Incidence, characteristics, management, and prognosis of the most common types of immune checkpoint inhibitor AKI

AKI Type	Incidence or % of Reported Cases	Risk Factors	Characteristics and Clinical Features	Management	Prognosis
ATIN	Incidence Overall 3.5%^81^–5.7%^26^ PD-1, 2.2%^24^ –5%^82^ PD-L1 <1%^83^–10%^82^ CTLA-4 2%^81^ PD-1/CTLA-4 combination 5%^81^ LAG-3 2%^84^	Older age^26^ Ipilimumab use^26^ Combined ICI use PPI use^28^ Presence of previous or concomitant extrarenal irAEs^20,50^	Hematuria Pyuria Mild proteinuria	Hold ICI and PPIs Hold NSAIDs, antimicrobials Corticosteroids Infliximab for refractory cases	Partial or complete recovery in most cases Rate of recurrence with rechallenge 16%–23%^19,20^
Pauci-immune GN/vasculitis	% of reported cases^23^ 27%	PD-1/PD-L1 pathway checkpoint inhibitor use	Negative ANCA serologies Nephritic syndrome	Corticosteroids	Dialysis in 25% of patients with vasculitis
MCD/FSGS	% of reported cases^23^ MCD 20% FSGS 7%	PD-1/PD-L1 pathway checkpoint inhibitor use	Nephrotic syndrome	Corticosteroids	>60% partial or complete remission
C3GN	% of reported cases^23^ C3GN 11%	PD-1/PD-L1 pathway checkpoint inhibitor use	Nephritic syndrome	Corticosteroids	Complete remission

ATIN, acute tubulo-interstitial nephritis; C3GN, C3 glomerulopathy; CTLA-4, cytotoxic T-lymphocyte–associated protein 4; ICI, immune checkpoint; irAE, immune-related adverse event; LAG-3, lymphocyte-activation gene 3; MCD, minimal change disease; NSAID, nonsteroidal anti-inflammatory drug; PD-1, programmed cell death protein 1; PD-L1, programmed death-ligand 1; PPI, proton pump inhibitor.

### General Principles of Diagnosis and Management

Early recognition and timely intervention are essential for optimal preservation of kidney function. The first step in management includes temporary discontinuation of the ICI agent along with discontinuation of PPIs and of medications associated with increased risk of developing AKI in general (nonsteroidal anti-inflammatory drugs and antibiotics). PPI use in particular has been associated with an odds ratio of 2.4 (1.79–3.23) for developing AKI in the context of ICI use in a large multicenter study.^20^ We recommend a kidney biopsy whenever feasible as several types of AKI lesions could coexist.^33^ This is in concordance with the latest National Comprehensive Cancer Network guidelines, which recommend considering a kidney biopsy for grade 2 AKI and above if there is no improvement within 5–7 days of discontinuation.^34^ Similarly, the updated American Society of Clinical Oncology guidelines require for diagnosis either a kidney biopsy or a typical presentation with sterile pyuria, peripheral eosinophilia, and no alternate diagnosis.^35^ If there is strong clinical suspicion for acute interstitial nephritis (AIN) or confirmation through a kidney biopsy, initial corticosteroid therapy such as prednisone 0.8–1.0 mg/kg per day dosing or equivalent with a maximum dose of prednisone 60–80 mg per day should be initiated promptly. In the absence of solid evidence-based data and a lack of consensus, the optimal duration of therapy and whether a slow or rapid taper of corticosteroids is the best treatment remain to be determined. Furthermore, it is unclear whether the half-life of the offending agent should dictate the steroid therapy duration. Personalization is important, and if a creatinine rise is noticed as the corticosteroid dose is being tapered, the dose should be promptly increased and the treatment course prolonged. Standard courses range between 6 and 8 weeks, with doses being tapered after the first week.^36^ In addition, other immunosuppression, including mycophenolate mofetil, infliximab, rituximab, and cyclophosphamide, can be used for patients with refractory cases that did not respond well to high-dose corticosteroids.^37^ In particular, the effectiveness of infliximab has been documented in a series of ten patients with relapsed ICI-AKI or patients failing to achieve complete response after primary therapy.^38^ The dose successfully used was 5 mg/kg as a one-time dose or monthly, as needed.

#### Noninvasive Diagnosis of ICI-AKI

**Figure 3:**
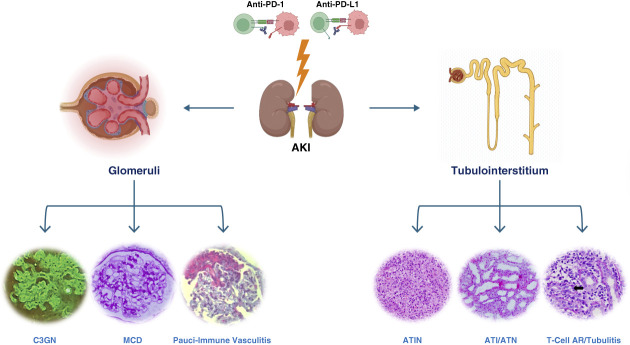
**Various kidney pathologies seen in ICI-AKI.** ATI, acute tubular injury; ATIN, acute tubulo-interstitial nephritis; ATN, acute tubular necrosis; C3GN, C3 glomerulopathy; MCD, minimal change disease; T-cell AR, T-cell–mediated acute rejection.

There have been attempts to use noninvasive tools such as biomarkers and imaging, such as positron emission tomography, to characterize and noninvasively diagnose ICI-AKI; however, their use has been limited to small series, and there is not enough evidence yet to recommend them for routine use.^39–42^ Biomarkers that have been investigated in small series and have shown some promise, including C-reactive protein; urine retinol binding protein-to-creatinine ratio;^43^ urine tumor necrosis factor-*α*, IL2, and IL10 levels^44^; soluble IL2 receptor *α*^45^; urine conserved cysteine motif ligand 9^40^; and urine monocyte chemoattractant protein-1.^46^ In particular, elevated urine levels of conserved cysteine motif ligand 9, an IFN-*γ*-induced chemokine involved in lymphocyte chemotaxis, showed strong association with ATIN in a prospectively enrolled cohort with pathologist-adjudicated histologic diagnoses.^40^ In a recent multicenter cohort which included 9 patients with ICI-AKI, there was a considerable increase in the mean standardized uptake value on 2-deoxy-2-[^18^F]fluoro-d-glucose positron emission tomography–computed tomography from baseline to the time of AKI in these patients compared with two groups of control patients.^47^ Figure 3 summarizes the noninvasive modalities that can be useful to diagnose ICI-AKI.

#### Prognosis of ICI-AKI

Reported complete renal recovery ranges between 40% and 65%.^19,20^ ICI rechallenge could be performed in most cases safely with or without low-dose glucocorticoids.^20,43^ The largest study to date of patients with ICI-AKI included 429 patients affected, of whom 121 were rechallenged with a rate of recurrence of 16% only. Importantly, 83% of the patients rechallenged were receiving low-dose glucocorticoids at the time of rechallenge.^20^ Recurrent AKI after rechallenging and stage 3 CKD at initiation increase the risk of CKD incidence and progression along with mortality risk. Patients with ICI-induced AKI have a higher mortality risk compared with those without AKI, and a meta-analysis of five studies conducted in various geographical locations and settings concluded to a 51% increase in risk of all-cause mortality (hazard ratio, 1.51; 95% confidence interval [CI], 1.07 to 2.14; I^2^=82.8%).^20,25,48–50^

## CAR-Ts

### Mechanism of Action and Preparation

**Figure 4:**
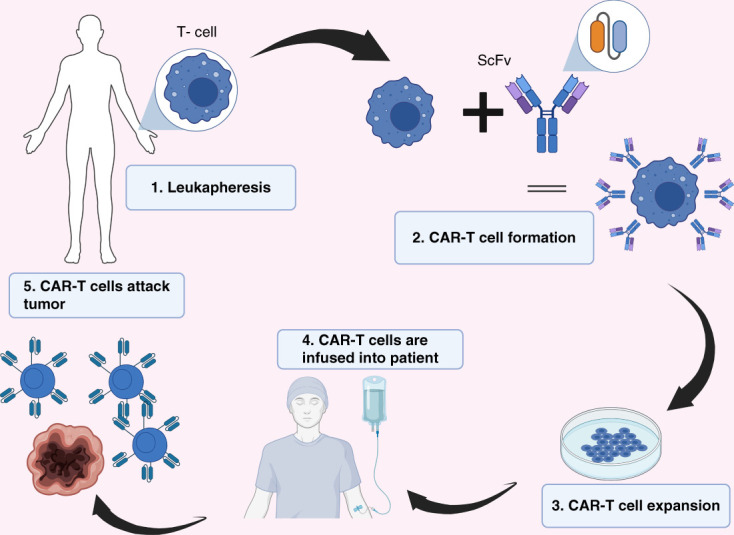
**Process of CAR T-cell production.** CAR-T, chimeric antigen receptors cell therapy; ScFV, single-chain variable fragment.

One of the mechanisms by which tumor cells avoid or escape immune surveillance is through loss of MHC expression on tumor cells and subsequent inadequate antigen presentation.^51^ Therefore, CAR T-cell construct has a single-chain variable fragment binding domain derived from a mAb that engages with the tumor antigen without the requirement for typical MHC restricted TCR signaling. The construct includes a costimulatory domain, which automatically provides the second activation signal required for activation and expansion of T-cells in the patient, initiating a cascade of cytotoxic signaling and leading to tumor lysis.^52,53^ CAR T-cell therapy preparation involves the *ex vivo* genetic manipulation of a patient’s own T lymphocytes, using either lentiviral or retroviral vectors, or nonviral gene transfer systems, to express engineered CARs specific for particular tumor targets^54–56^ (Figure 4). These reprogrammed CAR T-cells are then expanded and infused into the patient that would have been prepared for the therapy by receiving a lympho-depleting chemotherapy regimen. This lymphopenic environment causes release of homeostatic cytokines and consequent CAR T-cell expansion. Furthermore, antigen engagement leads to amplification of the CAR T-cells in the peripheral blood, which then migrate to tumor sites to detect and kill tumor cells expressing the corresponding antigen. This causes a chain reaction triggering further proliferation of CAR T-cells. Furthermore, tumor cell lysis leads to the release of tumor antigens, which activate the patient’s immune system to recruit non-CAR-T immune cells, thus eliciting stronger antitumor responses through a process known as cross-priming from epitope spreading. The process can be further enhanced by concomitant use of immunomodulatory agents.^57^ CAR T-cells can persist in the body for years and can maintain long-term disease control. Time from CAR T-cell collection to patient infusion currently takes around 3–4 weeks. Macrophages and NK cells can also be genetically altered using chimeric antigen receptor to specifically target and fix disease-related mutations, but are outside the scope of this review.^6^

### CAR T-Cells and the Kidney

Kidney side effects associated with the use of CAR T-cell therapy are common and include AKI and electrolyte abnormalities.^58,59^ The incidence of AKI is variable from one study to another and ranges between 5% and 30%.^58,60–62^ A recent systematic review found a pooled estimated incidence of AKI of 18.6% (95% CI, 14.3% to 23.8%; I^2^=77%) and a pooled estimated incidence of AKI needing KRT of 4.4% (95% CI, 2.1% to 8.9%; I^2^=61%).^63^ AKI occurs most frequently in the setting of CRS, a common complication of this therapy,^61,64^ and reported in more than 40% of the patients in published trials.^65^ CRS and immune effector cell–associated neurotoxicity syndrome another dreaded complication result from T-cell expansion. Contact with the target antigen induces CAR T-cell proliferation and cytokine production.^66^ The release of high concentrations of cytokines with a systemic inflammatory response leads to intravascular volume depletion due to vasodilation and increased vascular permeability with subsequent reduced kidney perfusion and AKI. CRS-related cardiomyopathy can also cause shock and subsequent AKI. This syndrome manifests with high fever, myalgias, and tachycardia within 1–14 days of CAR T-cell infusion with the rise in serum creatinine occurring shortly after.^67^ The severity of CRS is thought to be related to the CAR-T antigen target, use of lymphodepleting chemotherapy, such as fludarabine, and the magnitude of the inflammatory response. In addition, the type of costimulatory moiety used with possibly CD28 domains being more prone to trigger toxicity than 4-1BB domains could also play a role, even if use of different scales for grading CRS and neurotoxicity has made comparisons between trials using one type versus the other difficult.^68^ Of the available FDA-approved CAR T-cell products used (Supplemental Table 2), axicabtagene ciloleucel is associated with the most cases of CRS and subsequent AKI.^60,64^ Conversely, tisagenlecleucel is associated with reduced inflammation and lower rates of toxicity.^61,69,70^ Another side effect of CAR T-cell therapy, IEC-HLH, that is less common but serious and potentially fatal and arises from aggressive overactivation of the immune system, can also cause AKI through different mechanisms such as ATIN, acute tubular injury, and thrombotic microangiopathy.^71^ Finally, tumor lysis syndrome leading to AKI has also been noted with CAR T-cell therapy use.^72^ The injury tends however to be mild and fully recover.^60^ The different types of AKI related to CAR T-cell therapy are summarized in Table 2.

**Table 2:** Characteristics, management, and prognosis of the different types of CAR T-cell–associated AKI

Type of AKI	Clinical Features	Management	Prognosis
CRS-associated AKI	Flu-like symptoms, hypotension, capillary leak hypoxia, multi-organ failure neurotoxicity liver toxicity^85^	Corticosteroids, tocilizumab anakinra in CRS associated neurotoxicity ruxolitinib a JAK1/JAK2 inhibitor. Genetic manipulation of CAR T-cells engineered to express and secrete IL-1RA	Can be severe in approx. 46% of patients
TLS-associated AKI	Metabolic derangements–hyperkalemia, hyperuricemia, hyperphosphatemia, hypocalcemia^85^	Corticosteroids, Tocilizumab, KRT	Development of TLS during therapy portends a poorer prognosis
Immune-mediated AKI	Proteinuria and biopsy proven collapsing FSGS^86^	Conservative management with RAAS blockade, statin	Unknown Often the underlying disease foreshadows the prognosis
TMA	Rare hemolytic anemia, thrombocytopenia, elevated LDH^87^	Platelet transfusions Supportive therapy	Poor Some case reports with partial recovery of renal function and a limited course

CAR-T, chimeric antigen receptors cell therapy; CRS, cytokine release syndrome; GM-CSF, granulocyte macrophage-colony stimulating factor; IL-1RA, IL 1 receptor antagonists; JAK, Janus kinase; LDH, lactate dehydrogenase; RAAS, renin angiotensin aldosterone system; TLS, tumor lysis syndrome; TMA, thrombotic microangiopathy.

### General Principles of Management of AKI

Adequate oral or intravenous (IV) fluid administration plus dose reduction of lymphodepletion therapy when the kidney function is impaired can minimize the risk.^73^ In addition, CAR T-cell products with a minimal associated toxicity profile should be chosen as a therapeutic agent when feasible. CRS needs to be recognized early and managed aggressively with prompt initiation of steroids or preferably tocilizumab or anakinra as both IL-1 and IL-6 have been implicated as key factors in the genesis of adverse effects.^72,74,75^The use of anticytokines also preserves CAR T-cell function.^74^ Elevated biomarkers reliably predict the onset of CRS, but studies have not shown a correlation with the risk of AKI.^76^ Once AKI has set in, judicious management with IV fluids and pressor support where indicated may help improve patients outcomes with diuretics and IV albumin in cases with edema.^65^ Patients with tumor lysis syndrome should have vascular volume expansion and receive allopurinol or rasburicase. The management of patients with IEC-HLH includes steroids with anti-IL1 therapy as a first line and ruxolitinib a Janus kinase 1 and 2 inhibitor, in refractory cases.^71,73^ Furthermore, as IFN*γ* is increasingly being recognized as a key cytokine related to CAR T-cell therapy–mediated toxicities, several reports report efficacy of emapalumab, the IFN*γ*-blocking antibody, in addition to corticosteroids and multiple IL-directed treatments in patients with severe IEC-HLH and CRS.^77–79^ Finally, extracorporeal removal of cytokines using adsorption has been reported using Cytosorb, which is a device designed for the treatment of cytokine storm in the setting of overwhelming systemic inflammation seen with coronavirus disease 2019 infection.^80^ Its clinical utility, however, remains to be demonstrated.

#### Prognosis of CAR T-Cell–Associated AKI

AKI related to CRS is often mild; most patients do not need KRT and achieve a return to baseline creatinine within 1 month of onset of AKI.^60,73^ Dialysis, if required, portends a poorer prognosis.

## Conclusions

AIN constitutes the main mechanism for ICI-AKI and can only be confirmed through a kidney biopsy. Prompt discontinuation of the ICI and of any potential concomitant AIN-causing drugs with timely initiation of high-dose corticosteroids is necessary for the best outcome. Infliximab can be useful in severe or refractory cases. CAR T-cell therapy is growing in importance as an effective immunotherapy for cancer and causes AKI, primarily acute tubular injury and prerenal azotemia, through the unique side effects of CRS and IEC-HLH. Further studies are needed to prevent and diagnose these adverse events more effectively for an optimal use of these agents in patients with cancer at all levels of kidney health.

## Supplementary Material

## Supplemental Material

Supplemental Table 1. Available CAR T-cell therapies in clinical practice.

Supplemental Table 2. Common available immune checkpoint therapies in clinical practice.

## References

[1] Coley WB. The treatment of malignant tumors by repeated inoculations of erysipelas. Clin Orthop Relat Res. 1991;(262):3–11. doi:10.1097/00003086-199101000-00002 1984929

[2] Brentjens RJ Curran KJ. Novel cellular therapies for leukemia: CAR-modified T cells targeted to the CD19 antigen. Hematology Am Soc Hematol Educ Program. 2012;2012:143–151. doi:10.1182/asheducation-2012.1.143 23233573 PMC5536093

[3] Narayan V Barber-Rotenberg JS Jung IY, . PSMA-targeting TGFβ-insensitive armored CAR T cells in metastatic castration-resistant prostate cancer: a phase 1 trial. Nat Med. 2022;28(4):724–734. doi:10.1038/s41591-022-01726-1 35314843 PMC10308799

[4] Grupp SA Kalos M Barrett D, . Chimeric antigen receptor-modified T cells for acute lymphoid leukemia. N Engl J Med. 2013;368(16):1509–1518. doi:10.1056/NEJMoa1215134 23527958 PMC4058440

[5] Ansell SM. Fundamentals of immunology for understanding immunotherapy for lymphoma. Blood Adv. 2020;4(22):5863–5867. doi:10.1182/bloodadvances.2020002537 33232478 PMC7686892

[6] Kumar A Emdad L Das SK Fisher PB. Recent advances and progress in immunotherapy of solid cancers. Adv Cancer Res. 2024;164:111–190. doi:10.1016/bs.acr.2024.05.004 39306365

[7] Peng L Sferruzza G Yang L Zhou L Chen S. CAR-T and CAR-NK as cellular cancer immunotherapy for solid tumors. Cell Mol Immunol. 2024;21(10):1089–1108. doi:10.1038/s41423-024-01207-0 39134804 PMC11442786

[8] Moturi K Sharma H Hashemi-Sadraei N. Nephrotoxicity in the age of immune checkpoint inhibitors: mechanisms, diagnosis, and management. Int J Mol Sci. 2023;25(1):414. doi:10.3390/ijms25010414 38203586 PMC10778678

[9] Manohar S Jhaveri KD Perazella MA. Immunotherapy-related acute kidney injury. Adv Chronic Kidney Dis. 2021;28(5):429–437.e1. doi:10.1053/j.ackd.2021.07.006 35190109

[10] Shiravand Y Khodadadi F Kashani SMA, . Immune checkpoint inhibitors in cancer therapy. Curr Oncol. 2022;29(5):3044–3060. doi:10.3390/curroncol29050247 35621637 PMC9139602

[11] Granier C De Guillebon E Blanc C, . Mechanisms of action and rationale for the use of checkpoint inhibitors in cancer. ESMO Open. 2017;2(2):e000213. doi:10.1136/esmoopen-2017-000213 28761757 PMC5518304

[12] Mortezaee K. Immune escape: a critical hallmark in solid tumors. Life Sci. 2020;258:118110. doi:10.1016/j.lfs.2020.118110 32698074

[13] Sprangers B Leaf DE Porta C Soler MJ Perazella MA. Diagnosis and management of immune checkpoint inhibitor-associated acute kidney injury. Nat Rev Nephrol. 2022;18(12):794–805. doi:10.1038/s41581-022-00630-8 36168055

[14] Van Coillie S Wiernicki B Xu J. Molecular and cellular functions of CTLA-4. Adv Exp Med Biol. 2020;1248:7–32. doi:10.1007/978-981-15-3266-5_2 32185705

[15] Qureshi OS Zheng Y Nakamura K, . Trans-endocytosis of CD80 and CD86: a molecular basis for the cell-extrinsic function of CTLA-4. Science. 2011;332(6029):600–603. doi:10.1126/science.1202947 21474713 PMC3198051

[16] Ahmadzadeh M Johnson LA Heemskerk B, . Tumor antigen-specific CD8 T cells infiltrating the tumor express high levels of PD-1 and are functionally impaired. Blood. 2009;114(8):1537–1544. doi:10.1182/blood-2008-12-195792 19423728 PMC2927090

[17] Yokosuka T Takamatsu M Kobayashi-Imanishi W Hashimoto-Tane A Azuma M Saito T. Programmed cell death 1 forms negative costimulatory microclusters that directly inhibit T cell receptor signaling by recruiting phosphatase SHP2. J Exp Med. 2012;209(6):1201–1217. doi:10.1084/jem.20112741 22641383 PMC3371732

[18] Kong X Zhang J Chen S, . Immune checkpoint inhibitors: breakthroughs in cancer treatment. Cancer Biol Med. 2024;21(6):451–472. doi:10.20892/j.issn.2095-3941.2024.0055 38801082 PMC11208906

[19] Cortazar FB Kibbelaar ZA Glezerman IG, . Clinical features and outcomes of immune checkpoint inhibitor-associated AKI: a multicenter study. J Am Soc Nephrol. 2020;31(2):435–446. doi:10.1681/ASN.2019070676 31896554 PMC7003302

[20] Gupta S Short SAP Sise ME, . Acute kidney injury in patients treated with immune checkpoint inhibitors. J Immunother Cancer. 2021;9(10):e003467. doi:10.1136/jitc-2021-003467 34625513 PMC8496384

[21] Zhou P Gao Y Kong Z, . Immune checkpoint inhibitors and acute kidney injury. Front Immunol. 2024;15:1353339. doi:10.3389/fimmu.2024.1353339 38464524 PMC10920224

[22] Perazella MA Shirali AC. Immune checkpoint inhibitor nephrotoxicity: what do we know and what should we do? Kidney Int. 2020;97(1):62–74. doi:10.1016/j.kint.2019.07.022 31685311

[23] Kitchlu A Jhaveri KD Wadhwani S, . A systematic review of immune checkpoint inhibitor–associated glomerular disease. Kidney Int Rep. 2021;6(1):66–77. doi:10.1016/j.ekir.2020.10.002 33426386 PMC7783581

[24] Manohar S Kompotiatis P Thongprayoon C Cheungpasitporn W Herrmann J Herrmann SM. Programmed cell death protein 1 inhibitor treatment is associated with acute kidney injury and hypocalcemia: meta-analysis. Nephrol Dial Transplant. 2019;34(1):108–117. doi:10.1093/ndt/gfy105 29762725

[25] Xie W Xiao S Li X Huang J Li G Zhang Z. Incidence, mortality, and risk factors of acute kidney injury after immune checkpoint inhibitors: systematic review and meta-analysis of real-world evidence. Eur J Intern Med. 2023;115:88–95. doi:10.1016/j.ejim.2023.05.034 37263805

[26] Liu C Wei W Yang L, . Incidence and risk factors of acute kidney injury in cancer patients treated with immune checkpoint inhibitors: a systematic review and meta-analysis. Front Immunol. 2023;14:1173952. doi:10.3389/fimmu.2023.1173952 37313406 PMC10258324

[27] Chen P Zhu J Xu Y, . Risk factors of immune checkpoint inhibitor-associated acute kidney injury: evidence from clinical studies and FDA pharmacovigilance database. BMC Nephrol. 2023;24(1):107. doi:10.1186/s12882-023-03171-9 37087434 PMC10122540

[28] Seethapathy H Zhao S Chute DF, . The incidence, causes, and risk factors of acute kidney injury in patients receiving immune checkpoint inhibitors. Clin J Am Soc Nephrol. 2019;14(12):1692–1700. doi:10.2215/CJN.00990119 31672794 PMC6895474

[29] Liu F Wang Z Li X, . Comparative risk of acute kidney injury among cancer patients treated with immune checkpoint inhibitors. Cancer Commun. 2023;43(2):214–224. doi:10.1002/cac2.12396 PMC9926960 36528491

[30] Wang Q Moledina DG Sise ME. Immune checkpoint inhibitors and kidney disease. Curr Opin Nephrol Hypertens. 2022;31(5):449–455. doi:10.1097/MNH.0000000000000805 35894279

[31] Qin Q Patel VG Wang B, . Type, timing, and patient characteristics associated with immune-related adverse event development in patients with advanced solid tumors treated with immune checkpoint inhibitors. J Clin Oncol. 2020;38(15 suppl):e15160. doi:10.1200/JCO.2020.38.15_suppl.e15160

[32] Centanni M Moes DJAR Trocóniz IF Ciccolini J Van Hasselt JGC. Clinical pharmacokinetics and pharmacodynamics of immune checkpoint inhibitors. Clin Pharmacokinet. 2019;58(7):835–857. doi:10.1007/s40262-019-00748-2 30815848 PMC6584248

[33] Fenoglio R Cozzi M Del Vecchio G, . The need for kidney biopsy in the management of side effects of target and immunotherapy. Front Nephrol. 2023;3:1043874. doi:10.3389/fneph.2023.1043874 37675354 PMC10479613

[34] NCCN Clinical Practice Guidelines in Oncology (NCCN Guidelines®). Management of Immunotherapy-Related Toxicities, 2023. Accessed October 20, 2024. https://www.nccn.org/professionals/physician_gls/pdf/immunotherapy.pdf

[35] Schneider BJ Naidoo J Santomasso BD, . Management of immune-related adverse events in patients treated with immune checkpoint inhibitor therapy: ASCO guideline update. J Clin Oncol. 2021;39(36):4073–4126. doi:10.1200/JCO.21.01440 34724392

[36] Herrmann SM Perazella MA. Immune checkpoint inhibitors and immune-related adverse renal events. Kidney Int Rep. 2020;5(8):1139–1148. doi:10.1016/j.ekir.2020.04.018 32775813 PMC7403510

[37] Herrmann SM Abudayyeh A Gupta S, . Diagnosis and management of immune checkpoint inhibitor-associated nephrotoxicity: a position statement from the American Society of Onco-nephrology. Kidney Int. 2025;107(1):21–32. doi:10.1016/j.kint.2024.09.017 39455026

[38] Lin JS Mamlouk O Selamet U, . Infliximab for the treatment of patients with checkpoint inhibitor-associated acute tubular interstitial nephritis. Oncoimmunology. 2021;10(1):1877415. doi:10.1080/2162402X.2021.1877415 33643693 PMC7872057

[39] Moledina DG Wilson FP Pober JS, . Urine TNF-α and IL-9 for clinical diagnosis of acute interstitial nephritis. JCI Insight. 2019;4(10):e127456. doi:10.1172/jci.insight.127456 31092735 PMC6542610

[40] Moledina DG Obeid W Smith RN, . Identification and validation of urinary CXCL9 as a biomarker for diagnosis of acute interstitial nephritis. J Clin Invest. 2023;133(13):e168950. doi:10.1172/JCI168950 37395276 PMC10313360

[41] Qualls D Seethapathy H Bates H, . Positron emission tomography as an adjuvant diagnostic test in the evaluation of checkpoint inhibitor-associated acute interstitial nephritis. J Immunother Cancer. 2019;7(1):356. doi:10.1186/s40425-019-0820-9 31864416 PMC6925427

[42] Awiwi MO Abudayyeh A Abdel-Wahab N, . Imaging features of immune checkpoint inhibitor-related nephritis with clinical correlation: a retrospective series of biopsy-proven cases. Eur Radiol. 2023;33(3):2227–2238. doi:10.1007/s00330-022-09158-8 36255488 PMC9957799

[43] Isik B Alexander MP Manohar S, . Biomarkers, clinical features, and rechallenge for immune checkpoint inhibitor renal immune-related adverse events. Kidney Int Rep. 2021;6(4):1022–1031. doi:10.1016/j.ekir.2021.01.013 33912752 PMC8071627

[44] Farooqui N Zaidi M Vaughan L, . Cytokines and immune cell phenotype in acute kidney injury associated with immune checkpoint inhibitors. Kidney Int Rep. 2023;8(3):628–641. doi:10.1016/j.ekir.2022.11.020 36938084 PMC10014345

[45] Sise ME Wang Q Seethapathy H, . Soluble and cell-based markers of immune checkpoint inhibitor-associated nephritis. J Immunother Cancer. 2023;11(1):e006222. doi:10.1136/jitc-2022-006222 36657813 PMC9853261

[46] Martinez Valenzuela L Gómez-Preciado F Guiteras J, . Immune checkpoint inhibitors induce acute interstitial nephritis in mice with increased urinary MCP1 and PD-1 glomerular expression. J Transl Med. 2024;22(1):421. doi:10.1186/s12967-024-05177-9 38702780 PMC11069287

[47] Gupta S Green-Lingren O Bhimaniya S, . F18-FDG PET imaging as a diagnostic tool for immune checkpoint inhibitor–associated acute kidney injury. J Clin Invest. 2024;134(18):e182275. doi:10.1172/JCI182275 39115940 PMC11405038

[48] García-Carro C Bolufer M Bury R, . Acute kidney injury as a risk factor for mortality in oncological patients receiving checkpoint inhibitors. Nephrol Dial Transplant. 2022;37(5):887–894. doi:10.1093/ndt/gfab034 33547795

[49] Koks MS Ocak G Suelmann BBM, . Immune checkpoint inhibitor-associated acute kidney injury and mortality: an observational study. PLoS One. 2021;16(6):e0252978. doi:10.1371/journal.pone.0252978 34101756 PMC8186792

[50] Shimamura Y Watanabe S Maeda T Abe K Ogawa Y Takizawa H. Incidence and risk factors of acute kidney injury, and its effect on mortality among Japanese patients receiving immune check point inhibitors: a single-center observational study. Clin Exp Nephrol. 2021;25(5):479–487. doi:10.1007/s10157-020-02008-1 33471239

[51] Dhatchinamoorthy K Colbert JD Rock KL. Cancer immune evasion through loss of MHC class I antigen presentation. Front Immunol. 2021;12:636568. doi:10.3389/fimmu.2021.636568 33767702 PMC7986854

[52] Jayaraman J Mellody MP Hou AJ, . CAR-T design: elements and their synergistic function. EBioMedicine. 2020;58:102931. doi:10.1016/j.ebiom.2020.102931 32739874 PMC7393540

[53] Mazinani M Rahbarizadeh F. CAR-T cell potency: from structural elements to vector backbone components. Biomark Res. 2022;10(1):70. doi:10.1186/s40364-022-00417-w 36123710 PMC9487061

[54] Benmebarek MR Karches CH Cadilha BL Lesch S Endres S Kobold S. Killing mechanisms of chimeric antigen receptor (CAR) T cells. Int J Mol Sci. 2019;20(6):1283. doi:10.3390/ijms20061283 30875739 PMC6470706

[55] Alnefaie A Albogami S Asiri Y, . Chimeric antigen receptor T-cells: an overview of concepts, applications, limitations, and proposed solutions. Front Bioeng Biotechnol. 2022;10:797440. doi:10.3389/fbioe.2022.797440 35814023 PMC9256991

[56] Fischer JW Bhattarai N. CAR-T cell therapy: mechanism, management, and mitigation of inflammatory toxicities. Front Immunol. 2021;12:693016. doi:10.3389/fimmu.2021.693016 34220853 PMC8250150

[57] Conde E Vercher E Soria-Castellano M, . Epitope spreading driven by the joint action of CART cells and pharmacological STING stimulation counteracts tumor escape via antigen-loss variants. J Immunother Cancer. 2021;9(11):e003351. doi:10.1136/jitc-2021-003351 34810235 PMC8609946

[58] Gupta S Seethapathy H Strohbehn IA, . Acute kidney injury and electrolyte abnormalities after chimeric antigen receptor T-cell (CAR-T) therapy for diffuse large B-cell lymphoma. Am J Kidney Dis. 2020;76(1):63–71. doi:10.1053/j.ajkd.2019.10.011 31973908 PMC7311244

[59] Shaikh A. Immunotherapies and renal injury. Curr Opin Toxicol. 2022;31:100362. doi:10.1016/j.cotox.2022.100362 39086475 PMC11290437

[60] Gutgarts V Jain T Zheng J, . Acute kidney injury after CAR-T cell therapy: low incidence and rapid recovery. Biol Blood Marrow Transplant. 2020;26(6):1071–1076. doi:10.1016/j.bbmt.2020.02.012 32088364 PMC8375362

[61] Lee MD Strohbehn IA Seethapathy HS, . Acute kidney injury after the CAR-T therapy Tisagenlecleucel. Am J Kidney Dis. 2021;77(6):990–992. doi:10.1053/j.ajkd.2020.08.017 33098925 PMC8060348

[62] Ansari R Caimi P Lee HJ Chen Z Rashidi A. Renal outcomes after chimeric antigen receptor T-cell therapy: a single-center perspective. Nephrol Dial Transplant. 2022;37(9):1777–1779. doi:10.1093/ndt/gfac048 35218198

[63] Kanduri SR Cheungpasitporn W Thongprayoon C, . Systematic review of risk factors and incidence of acute kidney injury among patients treated with CAR-T cell therapies. Kidney Int Rep. 2021;6(5):1416–1422. doi:10.1016/j.ekir.2021.02.013 34013119 PMC8116758

[64] Lee DW Gardner R Porter DL, . Current concepts in the diagnosis and management of cytokine release syndrome. Blood. 2014;124(2):188–195. doi:10.1182/blood-2014-05-552729 24876563 PMC4093680

[65] Jhaveri KD Rosner MH. Chimeric antigen receptor T cell therapy and the kidney: what the nephrologist needs to know. Clin J Am Soc Nephrol. 2018;13(5):796–798. doi:10.2215/CJN.12871117 29523675 PMC5969488

[66] Penack O Koenecke C. Complications after CD19+ CAR T-cell therapy. Cancers. 2020;12(11):3445. doi:10.3390/cancers12113445 33228221 PMC7699604

[67] Perazella MA Shirali AC. Nephrotoxicity of cancer immunotherapies: past, present and future. J Am Soc Nephrol. 2018;29(8):2039–2052. doi:10.1681/ASN.2018050488 29959196 PMC6065079

[68] Cappell KM Kochenderfer JN. A comparison of chimeric antigen receptors containing CD28 versus 4-1BB costimulatory domains. Nat Rev Clin Oncol. 2021;18(11):715–727. doi:10.1038/s41571-021-00530-z 34230645

[69] Jaglowski S Hu ZH Zhang Y, . Tisagenlecleucel chimeric antigen receptor (CAR) T-cell therapy for adults with diffuse large B-cell lymphoma (DLBCL): real world experience from the center for international blood & marrow transplant research (CIBMTR) cellular therapy (CT) registry. Blood. 2019;134(suppl 1):766. doi:10.1182/blood-2019-130983

[70] Ying Z He T Wang X, . Parallel comparison of 4-1BB or CD28 Co-stimulated CD19-targeted CAR-T cells for B cell non-hodgkin’s lymphoma. Mol Ther Oncolytics. 2019;15:60–68. doi:10.1016/j.omto.2019.08.002 31650026 PMC6804784

[71] Fugere T Baltz A Mukherjee A, . Immune effector cell-associated HLH-like syndrome: a review of the literature of an increasingly recognized entity. Cancers. 2023;15(21):5149. doi:10.3390/cancers15215149 37958323 PMC10647774

[72] Porter DL Levine BL Kalos M Bagg A June CH. Chimeric antigen receptor-modified T cells in chronic lymphoid leukemia. N Engl J Med. 2011;365(8):725–733. doi:10.1056/NEJMoa1103849 21830940 PMC3387277

[73] Khan I Khan N Wolfson N Djebabria K Rehman MEU Anwer F. Safety of CAR-T cell therapy in patients with renal failure/acute kidney injury: focused review. Clin Hematol Int. 2023;5(2-3):122–129. doi:10.1007/s44228-023-00037-7 37010812 PMC10241763

[74] Namuduri M Brentjens RJ. Medical management of side effects related to CAR T cell therapy in hematologic malignancies. Expert Rev Hematol. 2016;9(6):511–513. doi:10.1080/17474086.2016.1183479 27139507 PMC5539903

[75] Garcia Borrega J Gödel P Rüger MA, . In the eye of the storm: immune-mediated toxicities associated with CAR-T cell therapy. Hemasphere. 2019;3(2):e191. doi:10.1097/HS9.0000000000000191 31723828 PMC6746039

[76] Teachey DT Lacey SF Shaw PA, . Identification of predictive biomarkers for cytokine release syndrome after chimeric antigen receptor T-cell therapy for acute lymphoblastic leukemia. Cancer Discov. 2016;6(6):664–679. doi:10.1158/2159-8290.CD-16-0040 27076371 PMC5448406

[77] McNerney KO DiNofia AM Teachey DT Grupp SA Maude SL. Potential role of IFNγ inhibition in refractory cytokine release syndrome associated with CAR T-cell therapy. Blood Cancer Discov. 2022;3(2):90–94. doi:10.1158/2643-3230.BCD-21-0203 35015687 PMC9245357

[78] Rainone M Ngo D Baird JH, . Interferon-γ blockade in CAR T-cell therapy-associated macrophage activation syndrome/hemophagocytic lymphohistiocytosis. Blood Adv. 2023;7(4):533–536. doi:10.1182/bloodadvances.2022008256 35917457 PMC9979758

[79] Scala JJ Eckrich MJ Lipak K, . Treatment strategies for progressive immune effector cell-associated hemophagocytic lymphohistiocytosis-like syndrome: case series. Haematologica. 2024;109(10):3439–3445. doi:10.3324/haematol.2023.284784 38813719 PMC11443363

[80] Stahl K Schmidt BMW Hoeper MM, . Extracorporeal cytokine removal in severe CAR-T cell associated cytokine release syndrome. J Crit Care. 2020;57:124–129. doi:10.1016/j.jcrc.2020.02.010 32113143

[81] Seethapathy H Herrmann SM Sise ME. Immune checkpoint inhibitors and kidney toxicity: advances in diagnosis and management. Kidney Med. 2021;3(6):1074–1081. doi:10.1016/j.xkme.2021.08.008 34939017 PMC8664750

[82] Zhou P Liu B Shen N, . Acute kidney injury in patients treated with immune checkpoint inhibitors: a single-center retrospective study. Ren Fail. 2024;46(1):2326186. doi:10.1080/0886022X.2024.2326186 38466161 PMC10930152

[83] Seethapathy H Zhao S Strohbehn IA, . Incidence and clinical features of immune-related acute kidney injury in patients receiving programmed cell death ligand-1 inhibitors. Kidney Int Rep. 2020;5(10):1700–1705. doi:10.1016/j.ekir.2020.07.011 33102962 PMC7569697

[84] Tawbi HA Schadendorf D Lipson EJ, . Relatlimab and nivolumab versus nivolumab in untreated advanced melanoma. N Engl J Med. 2022;386(1):24–34. doi:10.1056/NEJMoa2109970 34986285 PMC9844513

[85] Zhang Q Zu C Jing R, . Incidence, clinical characteristics and prognosis of tumor lysis syndrome following B-cell maturation antigen-targeted chimeric antigen receptor-T cell therapy in relapsed/refractory multiple myeloma. Front Immunol. 2023;14:1125357. doi:10.3389/fimmu.2023.1125357 37215107 PMC10192732

[86] Acharya R Horn B Zeng X Upadhyay K. Collapsing focal segmental glomerulosclerosis and acute kidney injury associated with chimeric antigen receptor T-cell (CAR-T) therapy: a case report. Kidney Med. 2021;3(6):1086–1090. doi:10.1016/j.xkme.2021.06.011 34939018 PMC8664733

[87] Wu MS Koirala A. Thrombotic microangiopathy following chimeric antigen receptor T-cell therapy. Clin Nephrol Case Stud. 2023;11(1):17–21. doi:10.5414/CNCS111045 36844260 PMC9948748

