# Supplementary material for: Acute Kidney Injury Associated with Novel Anticancer Therapies: Immunotherapy
Source: Kidney360. 2025 Feb 24;6(4):652–62. doi: 10.34067/KID.0000000749 (PMC12045508; doi:10.34067/KID.0000000749)
Supplement: Supplementary file 1 [file kidney360-6-652-s001.pdf]

ASN American Society of Nephrology | JASN Journal of the American Society of Nephrology | KIDNEY360 Accessing Our World From Every Angle | CJASN Clinical Journal of the American Society of Nephrology

ASN Journal Disclosure Form

As per ASN journal policy, I have disclosed any financial relationships or commitments I have held in the past 36 months as included below. I have listed my Current Employer below to indicate there is a relationship requiring disclosure. If no relationship exists, my Current Employer is not listed.

A. SH. Ali reports the following:
Employer: The Medical City , Baghdad

I understand that the information above will be published within the journal article, if accepted, and that failure to comply and/or to accurately and completely report the potential financial conflicts of interest could lead to the following: 1) Prior to publication, article rejection, or 2) Post-publication, sanctions ranging from, but not limited to, issuing a correction, reporting the inaccurate information to the authors' institution, banning authors from submitting work to ASN journals for varying lengths of time, and/or retraction of the published work.

Name: Ala A. SH. Ali
Manuscript ID: K360-2024-000910R1
Manuscript Title: Acute Kidney Injury associated with novel anti-cancer therapies: Immunotherapy
Date of Completion: February 19, 2025
Disclosure Updated Date: January 26, 2025

ASN
American Society of Nephrology
JASN
Journal of the American Society of Nephrology
KIDNEY360
Accessing Our World From Every Angle
CJASN
Clinical Journal of the American Society of Nephrology

ASN Journal Disclosure Form

As per ASN journal policy, I have disclosed any financial relationships or commitments I have held in the past 36 months as included below. I have listed my Current Employer below to indicate there is a relationship requiring disclosure. If no relationship exists, my Current Employer is not listed.

U. Anandh reports the following:
Employer: Amrita Institute of Medical Sciences and Research, Faridabad Delhi NCR

I understand that the information above will be published within the journal article, if accepted, and that failure to comply and/or to accurately and completely report the potential financial conflicts of interest could lead to the following: 1) Prior to publication, article rejection, or 2) Post-publication, sanctions ranging from, but not limited to, issuing a correction, reporting the inaccurate information to the authors' institution, banning authors from submitting work to ASN journals for varying lengths of time, and/or retraction of the published work.

Name: Urmila Anandh
Manuscript ID: K360-2024-000910R1
Manuscript Title: ACUTE KIDNEY INJURY ASSOCIATED WITH NOVEL ANTI-CANCER THERAPIES:IMMUNOTHERAPY.
Date of Completion: February 9, 2025
Disclosure Updated Date: November 25, 2024

ASN
American Society of Nephrology
JASN
Journal of the American Society of Nephrology
KIDNEY360
Accessing Our World From Every Angle
CJASN
Clinical Journal of the American Society of Nephrology

ASN Journal Disclosure Form

As per ASN journal policy, I have disclosed any financial relationships or commitments I have held in the past 36 months as included below. I have listed my Current Employer below to indicate there is a relationship requiring disclosure. If no relationship exists, my Current Employer is not listed.

W. Fung reports the following:
Employer: Prince of Wales Hospital, Hospital Authority

I understand that the information above will be published within the journal article, if accepted, and that failure to comply and/or to accurately and completely report the potential financial conflicts of interest could lead to the following: 1) Prior to publication, article rejection, or 2) Post-publication, sanctions ranging from, but not limited to, issuing a correction, reporting the inaccurate information to the authors' institution, banning authors from submitting work to ASN journals for varying lengths of time, and/or retraction of the published work.

Name: Winston Ws Fung
Manuscript ID: K360-2024-000910R1
Manuscript Title: Acute Kidney Injury associated with novel anti-cancer therapies: Immunotherapy
Date of Completion: January 27, 2025
Disclosure Updated Date: February 8, 2024

ASN American Society of Nephrology
JASN Journal of the American Society of Nephrology
KIDNEY360 Accessing Our World From Every Angle
CJASN Clinical Journal of the American Society of Nephrology

ASN Journal Disclosure Form

As per ASN journal policy, I have disclosed any financial relationships or commitments I have held in the past 36 months as included below. I have listed my Current Employer below to indicate there is a relationship requiring disclosure. If no relationship exists, my Current Employer is not listed.

S. Karam reports the following:
Employer: University of Minnesota; Consultancy: MediBeacon, Inc.; George Clinical; Honoraria: George Clinical; and Other Interests or Relationships: International Society of Nephrology; American Society of Onco-Nephrology; Societe Francophone de Nephrologie, Dialyse et Transplantation, National Kidney Foundation-Minnesota.

I understand that the information above will be published within the journal article, if accepted, and that failure to comply and/or to accurately and completely report the potential financial conflicts of interest could lead to the following: 1) Prior to publication, article rejection, or 2) Post-publication, sanctions ranging from, but not limited to, issuing a correction, reporting the inaccurate information to the authors' institution, banning authors from submitting work to ASN journals for varying lengths of time, and/or retraction of the published work.

Name: Sabine Karam
Manuscript ID: K360-2024-000910R1
Manuscript Title: Acute Kidney Injury associated with novel anti-cancer therapies: Immunotherapy
Date of Completion: January 28, 2025
Disclosure Updated Date: January 28, 2025

ASN
American Society of Nephrology
JASN
Journal of the American Society of Nephrology
KIDNEY360
Accessing Our World From Every Angle
CJASN
Clinical Journal of the American Society of Nephrology

ASN Journal Disclosure Form

As per ASN journal policy, I have disclosed any financial relationships or commitments I have held in the past 36 months as included below. I have listed my Current Employer below to indicate there is a relationship requiring disclosure. If no relationship exists, my Current Employer is not listed.

P. Mehta reports the following:
Employer: Amrita Institute of Medical Sciences and research centre; and Research Funding: Research funding to Institute for sponsored clinical trial; Tata Memorial Hospital and ImmunoACT.

I understand that the information above will be published within the journal article, if accepted, and that failure to comply and/or to accurately and completely report the potential financial conflicts of interest could lead to the following: 1) Prior to publication, article rejection, or 2) Post-publication, sanctions ranging from, but not limited to, issuing a correction, reporting the inaccurate information to the authors' institution, banning authors from submitting work to ASN journals for varying lengths of time, and/or retraction of the published work.

Name: Prashant Mehta
Manuscript ID: 4cfe3f46e24eb81d
Manuscript Title: Acute Kidney Injury associated with novel anti-cancer therapies: Immunotherapy
Date of Completion: February 6, 2025
Disclosure Updated Date: February 6, 2025

ASN American Society of Nephrology
JASN Journal of the American Society of Nephrology
KIDNEY360 Accessing Our World From Every Angle
CJASN Clinical Journal of the American Society of Nephrology

ASN Journal Disclosure Form

As per ASN journal policy, I have disclosed any financial relationships or commitments I have held in the past 36 months as included below. I have listed my Current Employer below to indicate there is a relationship requiring disclosure. If no relationship exists, my Current Employer is not listed.

S. Nair reports the following:
Employer: MIOT International; and Speakers Bureau: Pfizer India, Glaxosmithkline, Baxter India.

I understand that the information above will be published within the journal article, if accepted, and that failure to comply and/or to accurately and completely report the potential financial conflicts of interest could lead to the following: 1) Prior to publication, article rejection, or 2) Post-publication, sanctions ranging from, but not limited to, issuing a correction, reporting the inaccurate information to the authors' institution, banning authors from submitting work to ASN journals for varying lengths of time, and/or retraction of the published work.

Name: Sanjeev Nair
Manuscript ID: K360-2024-000910R1
Manuscript Title: Acute Kidney Injury associated with novel anti-cancer therapies: Immunotherapy
Date of Completion: January 28, 2025
Disclosure Updated Date: January 28, 2025
