# Supplementary material for: Acute Kidney Injury Associated with Novel Anticancer Therapies: Immunotherapy
Source: Kidney360. 2025 Feb 24;6(4):652–62. doi: 10.34067/KID.0000000749 (PMC12045508; doi:10.34067/KID.0000000749)
Supplement: Supplementary file 2 [file kidney360-6-652-s002.pdf]

Supplemental Table 1

The available CAR-T cell therapies in clinical practice.

Generic Name	Brand Name	Target Antigen	Indications
**Tisagenlecleucel**	KYMRIAH	CD19	B cell acute lymphoblastic leukemia (B-ALL)<br>B cell non-Hodgkin lymphoma (B-NHL)
**Axicabtagene Ciloleucel**	YESCARTA	CD19	B-NHL<br>Follicular lymphoma
**Brexucabtagene autoleucel**	TECARTUS	CD19	Mantle cell lymphoma<br>B-ALL
**Lisocabtagene Maraleucel**	BREYANZI	CD19	B-NHL
**Idecabtagene Vicleucel**	ABECMA	BCMA	Multiple myeloma
**Ciltacabtagene Autoleucel**	CARVYKTI	BCMA	Multiple myeloma

Abbreviations: BCMA-B cell maturation antigen

Supplemental Table 2: Common available Immune Checkpoint therapies in clinical practice

**Molecule**	**Class**	**Cellular Expression**	**Ligand**	**Common Indications**
Tremelimumab	CTLA-4	CD4+ T cells, CD8+ T cells, and Tregs[94]	CD80, CD86	HCC (with Durvalumab), NSCLC (with Durvalumab and platinum-based chemotherapy)
Ipilimumab	CTLA-4	CD4+ T cells, CD8+ T cells, and Tregs	CD80, CD86	Melanoma, RCC (with Nivolumab), NSCLC (with Nivolumad) ,Pleural mesothelioma (with Nivolumab)
Nivolumab	PD-1	T cells, DCs, NK cells, and B cells	PD-L1, PD-L2	NSCLC, RCC, SCLC (with Ipilimumab), HCC (with Ipilimumab) ,Melanoma (with Ipilimumab or alone), Esophageal squamous cell carcinoma (with Ipilimumab), Gastric and gastroesophageal cancer, HNSCC, Hodgkin's lymphoma,Urothelial carcinoma
Pembrolizumab	PD-1	T cells, DCs, NK cells, and B cells	PD-L1, PD-L2	Melanoma, Cutaneous squamous cell carcinoma, NSCLC, HNSCC, HCC, Urothelial carcinoma,

				Cervical cancer, Endometrial cancer, Gastric and gastroesophageal cancer, Hodgkin's lymphoma, Mediastinal B cell lymphoma, Merkel cell carcinoma, RCC, Triple negative breast cancer
Retifanlimab	PD-1	T cells, DCs, NK cells, and B cells	PD-L1, PD-L2	Merkel cell carcinoma
Cemiplimab	PD-1	T cells, DCs, NK cells, and B cells	PD-L1	Cutaneous squamous cell carcinoma, NSCLC, Basal cell carcinoma
Dostarlimab	PD-1	T cells, DCs, NK cells, and B cells	PD-L1, PD-L2	Endometrial cancer,
Atezolizumab	PD-L1	Tumor cells, tumor-infiltrating cells, and APCs	PD-L1	NSCLC, Urothelial carcinoma, SCLC, HCC (with Bevacizumab), Melanoma (with cobimetinib and vemurafenib)
Avelumab	PD-L1	Tumor cells, tumor-infiltrating cells, and APCs	PD-L1	Merkel cell carcinoma, Urothelial carcinoma, RCC

Durvalumab	PD-L1	Tumor cells, tumor-infiltrating cells, and APCs	PD-1	SCLC, NSCLC, Biliary tract cancer

Abbreviations:

1. **APCs**: Antigen-Presenting Cells
2. **CD80**: Cluster of Differentiation 80
3. **CD86**: Cluster of Differentiation 86
4. **CTLA-4**: Cytotoxic T-Lymphocyte Associated Protein 4
5. **DCs**: Dendritic Cells
6. **HCC**: Hepatocellular Carcinoma
7. **HNSCC**: Head and Neck Squamous Cell Carcinoma
8. **LAG3**: Lymphocyte Activation Gene 3
9. **MSI-H**: Microsatellite Instability-High
10. **NSCLC**: Non-Small Cell Lung Cancer
11. **PD-1**: Programmed Cell Death Protein 1
12. **PD-L1**: Programmed Death Ligand 1
13. **RCC**: Renal Cell Carcinoma
14. **SCLC**: Small Cell Lung Cancer
15. **TMB-H**: Tumor Mutational Burden-High
16. **Tregs**: Regulatory T Cells
